# Secular trends in consultations for asthma in early childhood, the 16 administrative regions of Morocco, 2004–2012

**DOI:** 10.1186/s12889-015-2262-8

**Published:** 2015-09-17

**Authors:** Mina Sadeq, Redouane Abouqal, Abdelilah ElMarnissi

**Affiliations:** Environmental Epidemiology Unit, National Institute of Hygiene, Ministry of Health, 27 Avenue Ibn Battota, BP 769 Rabat, Morocco; Laboratory of Biostatistiques, Clinical Research and Epidemiology, Faculty of Medicine and Pharmacy, University Mohammed V. Rabat, Rabat, Morocco; Service of Studies and Health Information, Direction of Planning and Financial Ressources, Ministry of Health, Rabat, Morocco

**Keywords:** Asthma epidemiology, Early Childhood, Secular trend, Morocco

## Abstract

**Background:**

Little is known about asthma trend in Morocco, particularly in early childhood. Furthermore, when dealing with asthma related environmental risk factors in Morocco, decision-making focus is in one region R9, while 16 regions make up the country. This work aims at studying 9-year trends in consultations for asthma in under-5 children in the 16 individual regions with respect to area and age group.

**Methods:**

Direct method use, based on the only available national data from the open access files of the ministry of health, standardizing data for three age groups (0–11 ; 12–23 and 24–59 months). We compared age-adjusted rates, stratified by area (urban and rural areas) within each region (Wilcoxon's signed ranks test), and between all regions emphasizing on R9. Secular trends are examined (Kendall's rank correlation test). We also compared directly standardized rates as a rate ratio for two study populations (that of R9 and any region with highest rates). We finally compared rates by age group in selected regions.

**Results:**

Secular increase in prevalence rates was shown in both urban and rural Morocco, particularly in urban areas of R10, R14, R16 and R5, and in rural areas of R14 and R16. In urban area of R10 (the highest age-adjusted prevalence rates area) the rates showed secular increase from 6.82 at 95 % CI = [6.44 to 7.19] per 1000 childhood population in 2004 to 20.91 at 95 % CI = [20.26 to 21.56] per 1000 childhood population in 2012 (P = 0.001). Rates were higher in urban than rural Morocco, particularly in R8, R9, R10, R14, R15 ; R6 was an exception. Rates in R10 were 1.63 higher than that in R9 in 2004 and rose to be 2.55 higher in 2012 ; rates in urban area of R14, about 3 times lower than that in R9 in 2004, increased to be similar in 2012. The highest-prevalence age group varied according to region and area.

**Discussion:**

The regions that worth decision making attention are the urban areas of R10 (the highest prevalence rates Moroccan area, showing continuous increase), of R9, of R14 and the rural area of R6. The rates in the urban area of R9 (a current continuous decision making focus) remained high but stable within the study period and less important than those in R10. Environmental factors (biological particules, non-biological particules or gazes) are suspected.The potential unavailability of treatment at regular basis at the primary health care centers may reduce frequency of consultations for asthma in early childhood : outpatients may consult only if asthma causes problems in an attempt to get free medicines ; chances of outpatients' follow-up by the primary health care center's physicians are therefore reduced and optimal asthma control is not achieved.

**Conclusion:**

Social, health care policy and environmental factors, to which decision-making has to be responsive, are suspected to be affecting both frequency of and time secular trend in consultations for asthma in early childhood in Morocco.

**Electronic supplementary material:**

The online version of this article (doi:10.1186/s12889-015-2262-8) contains supplementary material, which is available to authorized users.

## Background

According to WHO estimates [1], 235 million people suffer from asthma, and over 80 % of asthma deaths occurs in low and lower-middle income countries. In Morocco, a recent study (AIRMAG study) based on a questionnaire administered as a telephone survey, assessing asthma prevalence in the general population, carried out in 2008, showed that asthma prevalence in Morocco was about 3.9 % : 3,7 % in adults and 4,4 % in children [2–5]. From studies based on video questionnaires, “current wheeze” was highest in Morocco (12.9 %, 6–7 years old) in 2003 [6]. However, little is known about asthma secular trend in Morocco, and identifying asthma by a questionnaire remains a contentious issue [7, 8].

**Fig. 1:**
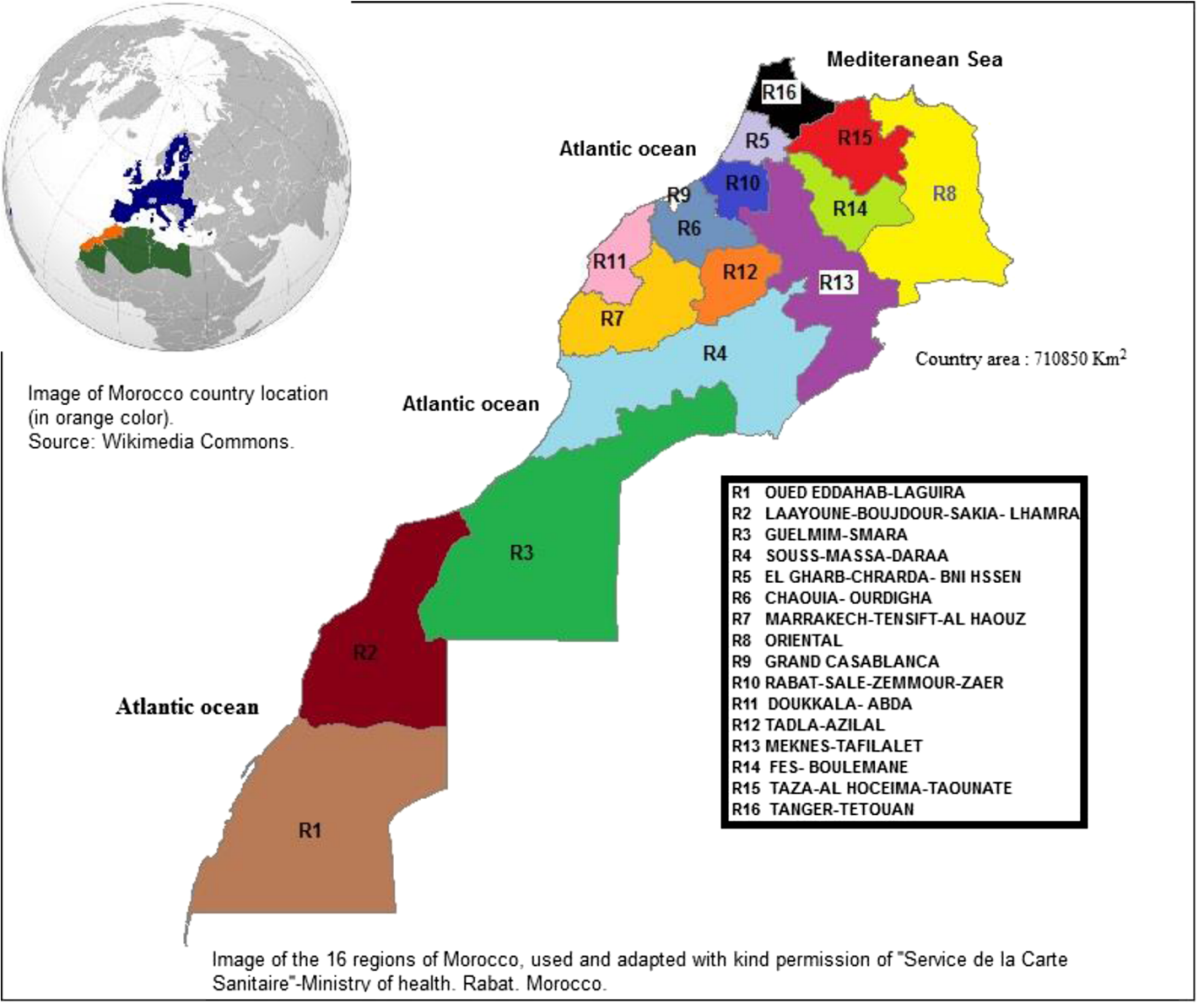
Map of Morocco illustrating the 16 administrative regions

According to WHO, asthma occurs in around 5–15 % in the pediadtic population [9]. Several studies show that as many as 50–80% of children who have asthma develop symptoms before their fifth birthdays [10]. In Morocco, asthma is a main chronic disease and a main cause of hospitalization in children. On the other hand, in Morocco, a country made up of 16 regions (Fig. 1), special attention is given to one region (Great Casablanca region or R9) as a focus of chronic respiratory diseases related to outdoor air pollution, particularly asthma. The reason behind this is the fact that it is a high population density area (density was 3288 habitants/km2 in 2008) and the largest most industrialized one in the country [11]. Thus several studies [12–14] were undertaken in R9 (two time series studies and one prevalence study). To our knowledge, a very few epidemiological studies on asthma were conducted in the other remaining 15 regions and none was about early childhood. We wonder if one of these 15 regions worth similar or more special attention than R9 while dealing with asthma in early childhood.

This work aims at investigating secular time trends in physician consultations for asthma, from 2004 to 2012 in children aged less than 5 years, in the 16 individual Moroccan regions, stratified by area (urban versus rural area) and standardizing prevalence related data for three age groups 0–11 months, 12–23 months and 24–59 months. This is to study related influencing factors, and to guide decision-making.

## Methods

### Study areas and study population

The country of Morocco is administratively subdivided into 16 regions. Each region consists of a few provinces and prefectures [15]. Primary health care is provided in primary health care centers available in both provinces and prefectures.

In this study, the 16 regions are identified as Region 1 (or R1), Region2 (or R2)… to Region 16 (or R16); we assigned R1, R2…R16 to each respective area in a image, depicting the 16 Moroccan regions, provided by the ''Service de la carte sanitaire''-Ministry of health, and that we adapt by adding colors and as text : ''Mediteranean sea'', ''Atlantic ocean'', the name of each region and the country area. A free image of the globe, from Wikimedia Commons, was also added to show the country of Morocco location) (Fig. 1).

In this study, we were interested in studying and comparing prevalence rates between regions and within regions stratifying each region by urban and rural areas. Children aged less than 5 years were the target population. They numbered 3,042,964 in 2004 and 2,909,537 children in 2012 [16].

### Source of data

#### Asthma diagnosis method and data collection at primary health care level

Primary health care in Morocco is a free of charge service. Medications are also provided for free for all age groups of population if the stock, provided by the Ministry of health, permits. Morocco had some 2510 primary health care centres in 2004, that reached 2759 ones in 2013 [16]. One may approach the nearest primary health care center where he/she lives ; they are not accepted in any other one but sent to their respective region's primary health care center. Overall, primary health care centers in Morocco do not have a laboratory or a X-ray device. The peak flow meter is the only available device to explore respiratory function in primary care. For less-than-five-year--age group, diagnosis usually consists of careful history and physical examination. Exploration of respiratory function is systematic when the child can perform it (around the age of 3 year). After consultation, physicians in primary care have to report all identified asthma cases by filling out a ''Bouclette'' (standardized form) whose data ends up at the Service of Studies and Health Information. Direction of planning and financial ressources. Ministry of health (DPRF/DPE/SEIS).

The patient can be a child with or without a history of asthma, and may consult a physician more than once (in case of asthma attack). Thus, in this study, we defined the number of all identified cases as the number of consultations for asthma in primary care, in children aged less than 5 years.

#### Asthma related data collection at national level

Asthma is a chronic disease that comes within the framework of a national Ministry of health program related to acute respiratory diseases [17]. The Service of Studies and Health Information yearly provides the number of all identified asthma cases (that is the number of consultations for asthma as defined previously) by group of age (children aged 0–11 months, 12–23 months and less than 5 years) detected each year, by region, by province/prefecture and by urban/rural area [16]. We calculated data for 24–59 months based on the previously cited one. These are the only available records in Morocco regarding asthma in early childhood. No ethics approval was required for this study.

Each year, a target population size (all the children aged less than 5 years living in each region) is provided by the High Commission for Planning (Maroc. HCP) and considered in all the programs of the Moroccan ministry of health including the previously cited program [16].

Data are available for the period of 2004 (edition 2005) to 2012 (edition 2013) [16] . We calculated age-adjusted prevalence rates of consultations for asthma by calendar year, by region and by urban/rural area (Additional files 1 and 2).

### Statistical analysis

**Fig. 2:**
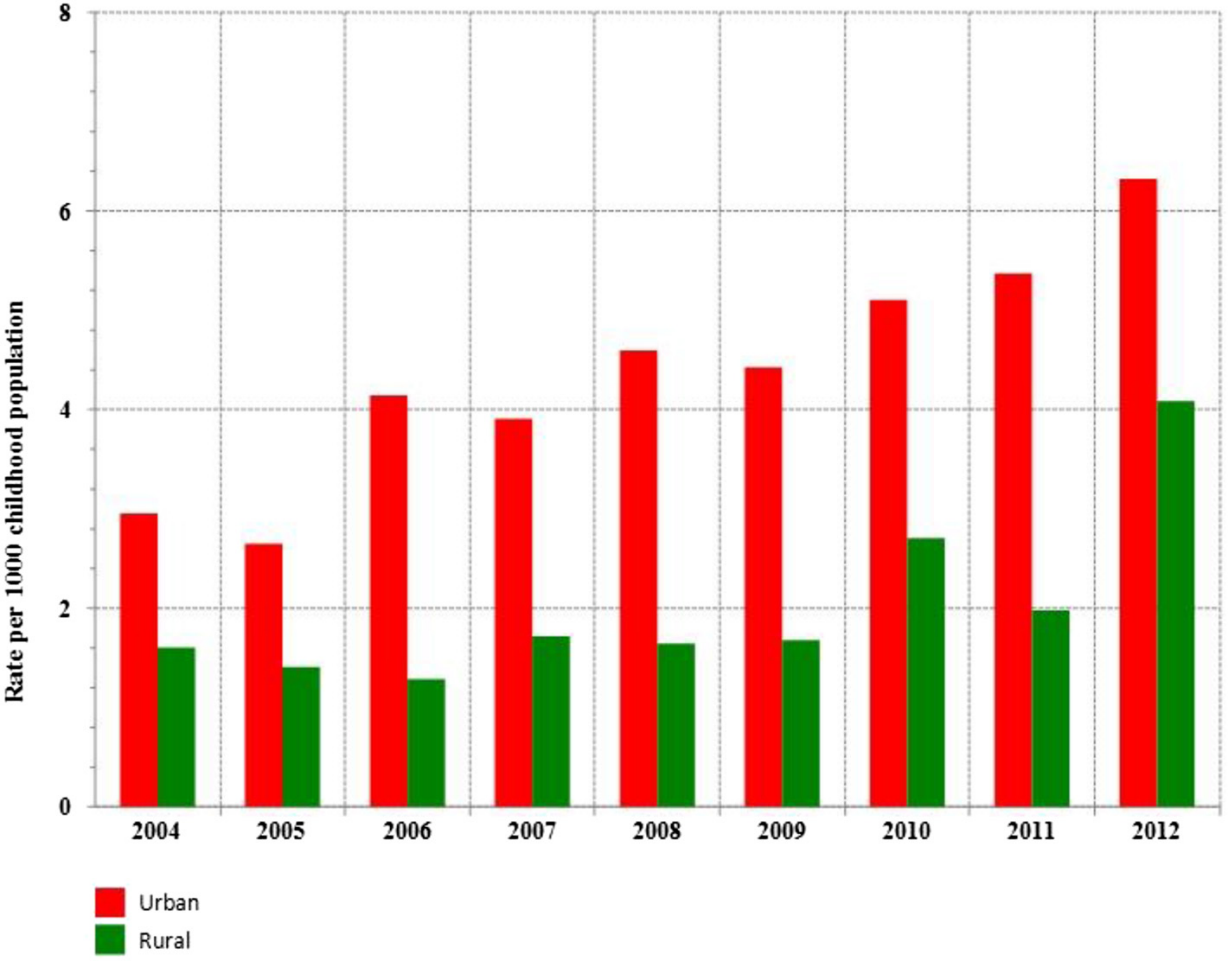
Prevalence rate of consultations for asthma, children aged less than five years, by area (urban/rural), Morocco, 2004–2012

Analysis of prevalence rates of consultations, all regions confounded, in both urban and rural areas, was first performed (Fig. 2). The age-adjusted prevalence rates of consultations for asthma were performated at 95 % confidence interval (CI) using the direct method : we standardized data for three age groups 0–11 months, 12–23 months and 24–59 months (Additional files 1 and 2), data about group sizes were identified for each studied year ; so were weights from a reference population which is the 2008 Moroccan children less than 5 years [16]. This was chosen since it is the middle year of the study period 2004–2012 : It is to be noted that a new population census whose data is still not published is that of September 2014, the previous population census was carried out 10 years ago (in 2004) which we chose not to base on in order to narrow differentials in target population data between year 2004 and year 2012. All rates are expressed per 1000 childhood population.

**Fig. 3:**
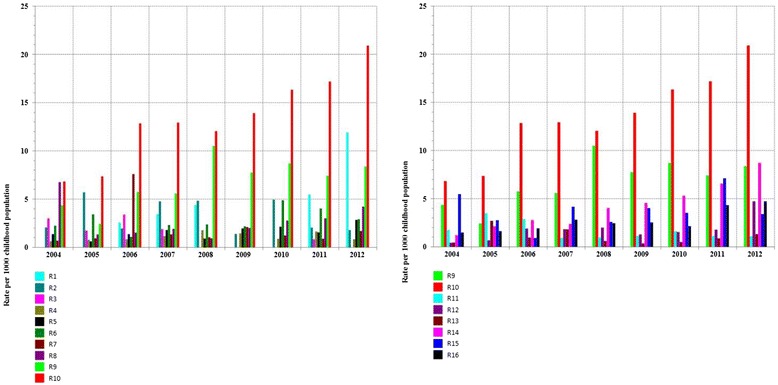
Age-adjusted prevalence rates of consultations for asthma, children aged less than five years, by administrative region (urban areas), Morocco, 2004–2012

**Fig. 4:**
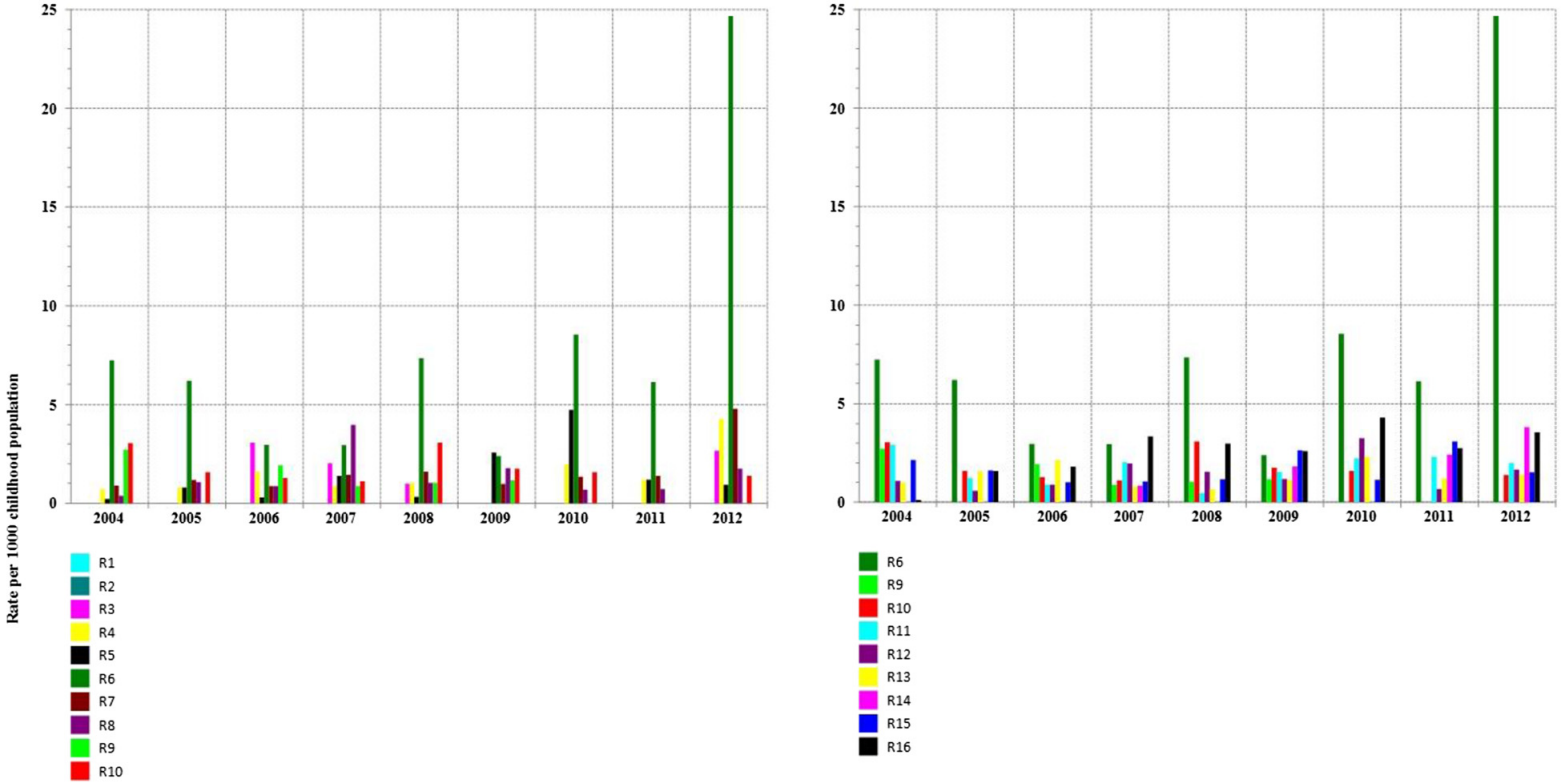
Age-adjusted prevalence rates of consultations for asthma, children aged less than five years, by administrative region (rural areas), Morocco, 2004–2012

We compared age-adjusted rates, stratified by area (urban and rural areas), between all regions taking R9 and the region with the highest rates as references (Figs. 3 and 4). We also compared age-adjusted rates, stratified by area (urban and rural areas), within each region using Wilcoxon's signed ranks test: p-Values (P) are provided. Variation of rates over time is also examined using an approximate two sided Kendall's rank correlation test (P is provided).

As we were interested in regions that may worth more attention than R9, we compared directly standardized rates (DSRs) as a rate ratio for two study populations (that of R9 and any region with higher rates). We finally compared rates by age group in selected regions using log transform.

All statistical analyses were done in StatDirect statistical software version 3.0.124 (StatsDirect Ltd. UK).

## Results

### Direct method of age-adjusted prevalence rates

Additional files 1 and 2 provide data on direct method for age-adjusted prevalence rate of consultations for asthma, in under-5 children for the 16 Moroccan regions, over a 9-year period (2004–2012), stratified by area (urban/rural). Prevalence rates are reported by age group (0–11 months, 12–23 months and 24–59 months) by region and by calendar year in urban areas (Additional file 1) and in rural areas (Additional file 2).

The highest age-adjusted prevalence rate of consultations for asthma was shown in R10 urban area (20.91 per 1000 childhood population) in 2012; the lowest age-adjusted prevalence rate was shown in R1 rural area (no case during the 9-year period). The overall trends by calendar year and by region are shown in Fig. 3 (urban areas) and Fig. 4 (rural areas).

### Prevalence rates comparison between urban and rural areas

Annual variations in prevalence rate of consultations for asthma in urban and rural Morocco (all regions confounded) were compared (Fig. 2): prevalence rates in urban Morocco were higher than those in rural Morocco (P = 0.002) and correlated to each others (P = 0.02). We also compared urban and rural areas prevalence rates within each region (Figs. 3 and 4). Age-adjusted prevalence rates were higher in the urban area than in the rural one in R8 (P = 0.027), R9 (P = 0.002), R10 (P = 0.004), R14 (P = 0.002), R15 (P = 0.004) and lower in R6 (P = 0.002). No difference was shown in the remaining regions.

In urban areas (Fig. 3), the highest age-adjusted prevalence rate of consultations for asthma was shown in R10 (20.91 per 1000 childhood population) in 2012, whereas in rural areas (Fig. 4), it was shown in R6 in 2012.

### Secular time trends

We observed an overall secular increase in both urban (P = 0.001) and rural Morocco (P = 0.01) (Fig. 2). In urban areas (Fig. 3), secular increase in age-adjusted prevalence rates was shown in R10 (P = 0.001), R14 (P = 0.0006), R16 (P = 0.009) and R5 (P = 0.036). In rural areas (Fig. 4), it was shown in R14 (P = 0,029) and R16 (P = 0,029) ; rates in R6 were high, but stable. In R14 and R16, prevalence rates showed secular increases in a similar way in both urban and rural areas, respectively. Prevalence rate in R9 (the decision-making focus) did not statistically change over time neither in the urban area nor in the rural one, whereas in the urban area of R10 (the highest age-adjusted prevalence rates region) the rates showed secular increase from 6.82 at 95 % CI = [6.44 to 7.19] per 1000 childhood population in 2004 to 20.91 at 95 % CI = [20.26 to 21.56] per 1000 childhood population in 2012 (P = 0.001).

### Comparison of age-adjusted prevalence rates between subgroups

There is no correlation between incidence rates in the urban areas of R10 and R9 despite of being adjacent regions (Fig. 1), while a correlation between rates is observed in the urban areas of the adjacent regions R10 and R5 (P = 0.012). Age-adjusted prevalence rates in R5, however, remained low (less than 3 per 1000 chilhood population). No statistical correlation was found between R9 and R6 or between R10 and R6, respectively, inspite of being adjacent regions.

We further investigated differences in age-adjusted prevalence rates between subgroups (R9 and any region with high rates). Comparison made between the urban areas of R10 and R9 showed that the standardized rate ratio (SDR) increased from SDR (R10/R9) = 1.63 at 95 % CI = [1.51 – 1.77] in 2004 to 2.55 at 95 % CI [2.42 – 2.69] in 2012. Whereas comparison between the urban areas of R9 and R14 showed that SDR (R9/R14) was 3.43 at 95 % CI = [2.89 to 4.07] in 2004 and changed to be similar in both regions SDR = 0.93 at 95 % CI = [0.86 to 1.00] in 2012.

### Comparison of prevalence rate by age group in selected regions

**Fig. 5:**
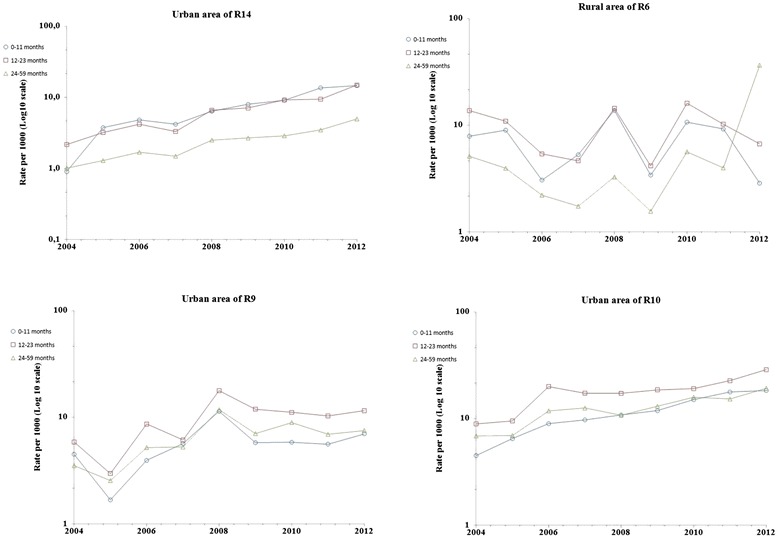
Prevalance rate (Log10 scale) of consultations for asthma, children <5 years, by age group, selected regions-Morocco, 2004–2012

**Fig. 6:**
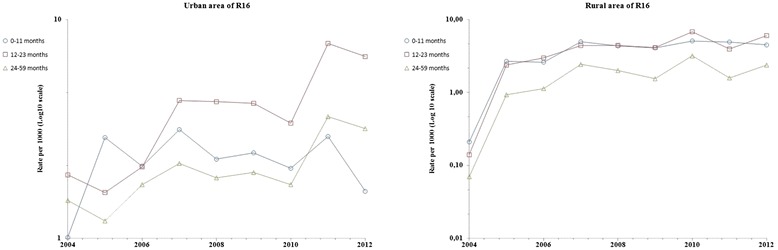
Prevalance rate (Log10 scale) of consultations for asthma, children <5 years, by age group, Region R16, 2004–2012

In order to further assess differences and similarities and to identify high-prevalence age group, we examined prevalence rates by age group from 2004 to 2012 in selected regions : the urban area of R10, that of R9, that of R14, the rural area of R6 (Fig. 5) and in R16 (both urban and rural areas) (Fig. 6). In the urban areas of R10 and R9, prevalence rates among 12–23 month_group were higher than those among 0–11 month_group and 24–59 month_group (P = 0.002 in both R10 and R9), showing an upward trend between 2004 and 2012 in R10 and similar fluctuations (pics and declines) in all age groups in R9. In R14, prevalence rates in both 0–11 month_group and 12–23 month_group were higher than those among 24–59 month_group (P = 0.002). A more steepy rate increase was observed in 0–11 month_group and 12–23 month_group than in 24–59 month_group, during the study time period. In the rural area of R6, annual similar fluctuations (pics and declines) were shown in all age groups ; prevalence rates among 12–23 month_group and 24–59 month_group were higher than those among 0–11 month_group (P = 0.006).

Prevalence rates among 12–23 month_group were higher than those in 0–11 month_group and in 24–59 month_group (P < 0.01) in the urban area of R16. In the rural area of this region, rates increased in a similar way for all age group, they were higher in 0–11 month_group and 12–23 month_group than in 24–59 month_group (P = 0.002). Even if an increase is shown in both areas, annual fluctuations were not similar in these two areas of R16.

## Discussion

**Fig. 7:**
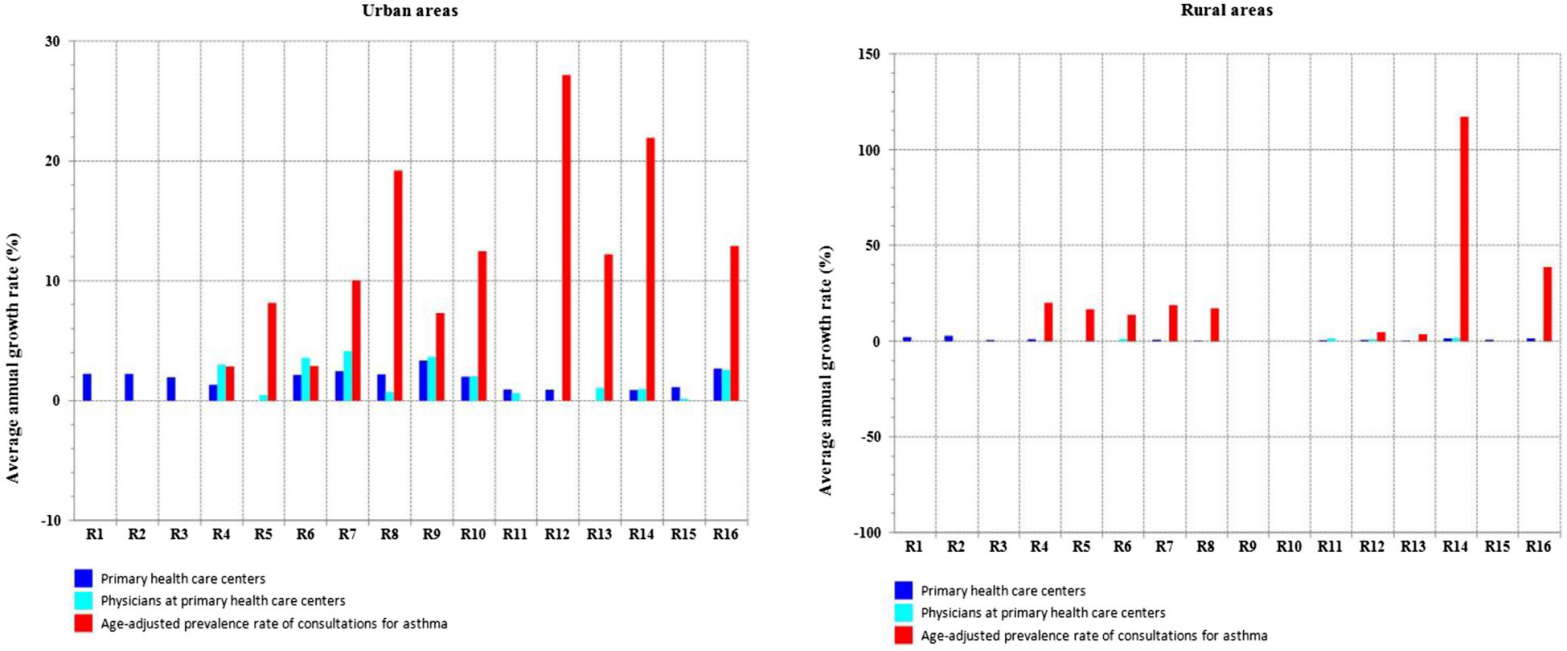
Average annual growth rate (%) in primary health care centers number (between 2004 and 2013), in physicians number at primary health care centers (between 2004 and 2013), and in prevalence rates of consultations for asthma (between 2004 and 2012), by administrative region, Morocco. Note: Data related to the number of primary health care centers, and that of physicians at these facilities, at region-level, corresponding to the years 2004 and 2013, are available [16]

This is the first report estimating trends in asthma in under-5 children at national level, based on the only accessible data related to asthma in Morocco, and identifying ''an asthma case'' by a physician diagnosis rather than by a telephone questionnaire (see Methods). If we consider a prevalence rate of consultations for asthma as an approximate surrogate measure of asthma prevalence rate, comparisons with other previous studies may be made. In the current study, the overall prevalence rates of consultations for asthma have increased between 2004 and 2012, which is consistent with expectations/findings about asthma prevalence increase reported in other studies [3, 6, 18]. Pearce *et al*. reported stronger increase in North Africa (Morocco, Algeria and Tunisia) than in English language countries and Western Europe [18]. The prevalence rates in the current study were more higher in urban areas than in rural ones, which is also consistent with findings of previous studies estimating asthma prevalence, and that were conducted in AIRMAG study (Tunisia, Morocco, Algeria) [3], in Africa [19] and elsewhere [14, 20]. Considering the under-5 age group, rates found in the current study can not be compared to those of the International Survey of Asthma and Allergy in Children (ISAAC) [14] which considered participants of different age groups (6–7 years old and 13–14 years old) and were restricted only to urban areas as to Morocco. However, the recent AIRMAG study, carried out in 2008, allowed comparison as it considered a random population sample (aged under sixteen) and rate related to under-5 children was provided [4]. There is a major difference between prevalence rate of consultations for asthma, in under-5 children (0.32 %, all regions confounded) reported in the current study in 2008 (it reached 0.53 % in 2012) and asthma prevalence rates (about 4.5 %) reported in AIRMAG study in the under-five children [4]. This may be explained by many factors. AIRMAG survey [4] showed that most participants consulted a private community practice for their asthma and were followed by a specialist, and that in Morocco, more children attended private health care providers (76.7 % in private community practice; 5.5 % in private hospital clinic) and consulted specialists than in the other two countries (Algeria and Tunisia). In contrast, the current study concerned only the public sector (primary health care centers : see Methods) where general pratictionars are available; a specialist may or may not be available (disparities regarding specialists availability still exist between the Moroccan administrative regions), which is challenging as to the delivery of asthma related medicines in children of this special group of age (under-5 children) [21]; the current study concerned participants whose parents often have no health insurance, can not afford to consult a private specialist, to regularly buy asthma related medicines, or to own a peak flow meter for use at home. Besides, although in Morocco medications at primary health care centers are free of charge, the available heath center's share often does not meet the needs of the population, which may reduce frequency of physician consultations for asthma : the outpatient may consult only to get free medicines or a free prescription from the health care center's physicians. The AIRMAG survey [4] showed that the frequency of physician consultations for asthma was higher in Algeria, with 42.9 % of children consulting a physician at least once a month (as public community care providers are most used and access to them is free in Algeria), and that in Morocco over a third of children only consulted once or twice a year or less. Prevalence rate of consultations for ashma in primary health care in Morocco may vary over time and place due to health care policy factors (availability, at region-level, of primary health care centers, of health practionners, and of medicines). The Fig. 7 showed that these two first factors are not associated with limited access to primary health care centers : different patterns were observed in Fig. 7, showing discrepencies at both region and area levels ; no statistical correlation was observed (P > 0.05) between the three annual variables. The unavailability of treatment at regular basis may be one explanation. Outpatients may consult only if asthma causes problems in an attempt to get free medicines: chances of outpatients' follow-up by the primary health care center's physicians are therefore reduced and optimal asthma control is not achieved. The AIRMAG survey [3, 4] showed that asthma control was best in Tunisia and worst in Morocco (p inter-country difference value = 0.03). The low frequency of consultations for asthma, in early childhood, in both the public sector (the current study) and the private one (AIRMAG survey), should the outpatient regularly have to buy medecines, may reveal that dealing with a chronic disease such as asthma still overtakes the financial capacities of the Moroccan outpatient. This may guide heath care policy regarding asthma management in early childhood in Morocco.

Prevalence of consultations for ashma in primary health care in Morocco may also vary over time and place due to environmental factors (triggers that urge outpatients to consult a physician). Financial ressouces were being allocated exclusively to one Moroccan region (R9) to carry out, since 1999, three consecutive epidemiological studies in R9 [12–14], whereas no similar study was undertaken in one of the remainning 15 regions so far. The current study revealed that the rates in both urban and rural Morocco are increasing and are statistically correlated to each other (Fig. 2), which can not be attributed only to lifestyle westernization. Environmental factors are suspected. Overall, the highest age-adjusted prevalence rates of consultations for asthma were reported in the urban area of R10 (20.91 per 1000 childhood population in 2012), overtaking those in R9 even if the average annual growth rate in primary health care centers and in physicians numbers was more important in R9 than in R10 (Fig. 7; urban areas). The lowest age-adjusted prevalence rates were reported in the rural area of R1 (no case during the 9-year period). Even if the average annual variation in prevalence rates of consultations for asthma in some regions (R7, R8, R12 and R13) were important (Fig. 7; urban areas), age-adjusted prevalence rates observed each year in these regions remained low and did not exceed 5 per 1000 childhood population (Fig. 3 and Additional file 1). Thus the Moroccan regions that worth decision making's attention are located in the northern Atlantic coast (R9, R10, R16 and R5) and in the central north (R14 and R6) of Morocco. In the current study, the highest age-prevalence rate group (Figs. 5 and 6) varied according to region and to area, which supports environmental triggers hypothesis. In the urban area of R9 (the largest most industrialized region in the country), the similar annual fluctations among all studied age groups (Fig. 5) may suggest potential different causal mechanisms (biological particules, non-biological particules or gazes) or most likely different levels of asthma triggers from one source. Considering the time period of this study (from 2004 to 2012), the urban area of R10 has always been the highest prevalence rate area; furthermore, rates in the urban area of R10 were statistically correlated to those in that of R5, which raises a question as to ''whether the causes of asthma in R10 affect R5''. The urban area of R10 is a fertile area for further investigation by means of analytic methods. Due to continuous rates increase, R16 has to be monitored closely and urban area of R14 requires further research for underlying risk factors. The multimodal (many pics) distribution in the rural area of R6 (Fig. 5) may suggest many different causal mechanisms. It is an agricultural mining region (pesticides, phosphate, zinc, lead, marble quarry, gravel quarries) whose both air and soil pollutants are to be considered as risk factors in future asthma related etiologic studies.

Our study has limitations. Gender related data is not available, which would have given more information regarding prevalence rates distribution by gender. Data related to consultations for asthma, used in the current study, include the public sector rather than the private one. If available, figures from this later would be added to the numerator, which would probably raise prevalence rates.

## Conclusion

The rates of asthma reported cases in under-5 children, within 2004–2012, have been increasing in Morocco. They were more important in the urban area than the rural one. The regions that worth decision making attention are the urban areas of R10 (the highest prevalence rates Moroccan area, showing continuous increase), of R9, of R14 and the rural area of R6. The rates in the urban area of R9 (a current continuous decision making focus) remained high but stable within the study period and less important than those in R10. Social, health care policy and environmental factors are suspected to be affecting both frequency of and secular time trend in consultations for asthma at primary health care centers. The highest prevalence rate group varies according to region and area, in Morocco. These findings would guide to adopt appropriate health policy, at region-level, regarding setting priorities and managing asthma in early childhood in Morocco.

## Additional files

Additional file 1:
**Direct method for age-adjusted prevalence rate of consultations for asthma in under-5 children, urban areas, Morocco: 9-year trends (2004-2012).** (PDF 45 kb) Additional file 2:
**Direct method for age-adjusted prevalence rate of consultations for asthma in under-5 children, rural areas, Morocco: 9-year trends (2004-2012).** (PDF 44 kb)

## Abbreviations

DSR: Directly standardized rate
P: p-Value
R: Region
