# Supplementary material for: Secular trends in consultations for asthma in early childhood, the 16 administrative regions of Morocco, 2004–2012
Source: BMC Public Health. 2015 Sep 17;15:905. doi: 10.1186/s12889-015-2262-8 (PMC4574130; doi:10.1186/s12889-015-2262-8)
Supplement: Additional file 1: — Direct method for age-adjusted prevalence rate of consultations for asthma in under-5 children, urban areas, Morocco: 9-year trends (2004-2012). (PDF 45 kb) [file 12889_2015_2262_MOESM1_ESM.pdf]

Additional File 1 : Direct method for age-adjusted prevalence rate of consultations for asthma in under-5 children, urban areas, Morocco : 9-year trends (2004-2012)

Age group (month)	2004	2005	2006	2007	2008	2009	2010	2011	2012
Region 1									
AdjR (95% CI)	0	*	2.56(1.56-3.57)	3.43(2.31-4.55)	4.39(3.18-5.61)	*	*	5.48 (4.29-6.66)	11.91 (10.10-13.71)
0-11	0 (0 – 3.77)	0 (0-2.59)	1.30 (0.16-4.70)	0 (0-2.22)	0 (0-2.07)	0 (0-1.92)	0 (0-1.65)	2.08 (0.68-4.86)	0.91 (0.11-3.30)
12-23	0 (0 – 3.83)	1.41 (0.17-5.10)	5.23 (2.26-10.31)	3.63 (1.33-7.91)	0.56 (0.01-3.15)	0 (0-1.93)	4.95 (2.47-8.85)	18.00 (13.03-24.24)	29.89 (23.06-38.09)
24-59	0 (0 – 1.32)	0.66 (0.14-1.94)	2.47 (1.26-3.70)	4.16 (2.81-5.94)	6.26 (4.63-8.27)	1.91 (1.09-3.10)	0 (0-0.39)	3.42 (2.37-4.77)	10.34 (8.41-12.58)
Region 2									
AdjR (95% CI)	2.06 (1.49-2.63)	5.70 (4.80-6.59)	1.96 (1.44-2.48)	4.77 (3.97-5.57)	4.83 (4.04-5.61)	1.38 (0.97-1.86)	4.94 (4.15-5.74)	2.05 (1.54-2.55)	1.80 (1.32-2.27)
0-11	6,70 (4.62-9.37)	3.79 (2.32-5.83)	3.27 (1.94-5.18)	0.70 (0.19-1.79)	3.55 (2.20-5.43)	0.81 (0.26-1.90)	4.78 (3.20-6.87)	2.27 (0.55-2.50)	0.83 (0.27-1.94)
12-23	0,21 (0.01-1.15)	8.00 (5.76-10.81	1.10 (0.40-2.39)	6.32 (4.42-8.74)	5.44 (3.72-7.67)	1.15 (0.46-2.36)	5.80 (4.04-8.07)	2.23 (1.22-3.75)	1.50 (0.69-2.86)
24-59	1,15 (0.66-1.87)	5.58 (4.51-6.82)	1.81 (1.22-2.58)	5.60 (4.54-6.82)	5.04 (4.06-6.18)	1.64 (1.12-2.33)	4.72 (3.76-5.84)	2.24 (1.61-3.04)	2.21 (1.58-2.99)
Region 3									
AdjR (95% CI)	3.00 (2.41-3.58)	1.74 (1.26-2.22)	3.40(2.76-4.04)	1.88 (1.41-2.35)	*	*	*	0.83(0.52-1.15)	*
0-11	3,13 (1.91- 4.83)	0.84 (0.27-1.97)	1.80 (0.90-3.23)	1.27 (0.55-2.51)	0.16 (0.004- 0.87)	0.30 (0.04-1.10)	1.11 (0.45-2.29)	0 (0-0.57)	0.32 (0.04-1.16)
12-23	4,26 (2.78-6.25)	2.04 (1.05-3.56)	5.12 (3.48-7.27)	2.73 (1.59-4.37)	0.79 (0.26-1.84)	0 (0-0.57)	0.16 (0.004-0.90)	0 (0-0.58)	0.65 (0.18-1.65)
24-59	2,57 (1.94-3.33)	1.93 (1.33-2.71)	3.36 (2.60-4.29)	1.81 (1.26-2.50)	0.77 (0.44-1.25)	0.09 (0.01-0.34)	0.25 (0.08-059)	1.35 (0.89-1.96)	0.87 (0.52-1.38)
Region 4									
AdjR (95% CI)	0.65 (0.52-0.79)	0.78 (0.63-0.94)	0.84 (0.69-1.00)	1.17 (0.99-1.35)	1.78 (1.56-2.00)	1.45 (1.26-1.65)	0.88 (0.73-1.04)	1.65 (1.44-1.85)	0.84 (0.70-0.98)
0-11	0,51 (0.27-0.88)	0.75 (0.46-1.19)	0.20 (0.07-0.47)	1.29 (0.89-1.81)	2.28 (1.74-2.93)	1.70 (1.26-1.65)	0.35 (0.16-0.66)	1.46 (1.04-2.00)	1.74 (1.28-2.33)
12-23	0,38 (0.17-0.72)	0.92 (0.58-1.40)	0.24 (0.09-0.53)	1.42 (0.99-1.96)	2.92 (2.30-3.75)	2.50 (1.94-3.17)	1.56 (1.12-2.13)	2.66 (2.07-3.35)	0.77 (0.58-1.00)
24-59	0,77 (0.60-0.99)	0.75 (0.57-0.97)	1.20 (0.98-1.46)	1.06 (0.85-1.30)	1.30 (1.08-1.56)	1.08 (0.88-1.31)	0.84 (0.66-1.06)	1.41 (1.18-1.97)	0.60 (0.45-0.77)
Region 5									
AdjR (95% CI)	1.37 (1.11-1.63)	0.62 (0.45-0.80)	1.37(1.10-1.63)	1.80 (1.50-2.10)	0.91 (0.70-1.12)	1.96 (1.65-2.27)	2.13 (1.80-2.46)	1.55 (1.26-1.85)	2.85 (2.48-3.22)
0-11	2,66 (1.93-3.53)	1.10 (0.63-1.78)	3.15 (2.31-4.19)	0.98 (0.55-1.62)	0.65 (0.31-1.19)	3.71 (1.13-2.49)	1.64 (1.06-2.42)	1.61 (1.02-2.42)	4.01 (3.04-5.19)
12-23	1,27 (0.78-1.96)	0.41 (0.15-0.90)	1.41 (0.87-2.16)	1.18 (0.70-1.87)	1.56 (1.00-2.32)	3.64 (2.75-4.71)	1.71 (1.12-2.51)	1.55 (0.97-2.34)	2.62 (2.11-3.22)
24-59	0,99 (0.71-1.33)	0.54 (0.35-0.80)	0.78 (0.54-1.08)	2.26 (1.85-2.74)	0.79 (0.56-1.09)	1.51 (1.18-1.90)	2.42 (1.98-2.92)	1.54 (1.18-1.97)	2.55 (2.10-3.07)
Region 6									
AdjR (95% CI)	2.25 (1.90-2.61)	3.42 (3.00-3.85)	1.09(1.28-1.75)	2.31 (1.96-2.66)	2.36 (2.00-2.71)	2.18 (1.84-2.51)	4.87 (4.35-5.39)	4.03 (3.55-4.50)	2.92 (2.57-3.27)
0-11	1.03 (0.58-1.70)	1.99 (1.31-2.90)	1.01 (0.55-1.70)	2.05 (1.37-2.94)	1.68 (1.07-2.49)	2.75 (1.96-3.74)	4.81 (3.72-6.12)	2.67 (1.88-3.67)	4.08 (3.11-5.27)
12-23	5.42 (4.26-6.79)	6.45 (5.17-7.96)	1.67 (1.06-2.50)	3.84 (2.88-5.00)	5.33 (4.20-6.68)	4.21 (3.22-5.41)	9.31 (7.76-11.08)	6.88 (5.57-8.41)	3.31 (2.72-4.00)
24-59	1.64 (1.27-2.10)	2.92 (2.43-3.48)	0.93 (0.66-1.26)	1.91 (1.52-2.36°	1.63 (1.28-2.03)	1.36 (1.04-1.75)	3.48 (2.94-4.10)	3.56 (3.00-4.18)	2.43 (2.02-2.90)
Region 7									
AdjR (95% CI)	0.69 (0.54-0.84)	0.94 (0.76-1.11)	7.59 (7.09-8.09)	1.35 (1.14-1.55)	1.05 (0.87-1.23)	2.10 (1.84-2.35)	1.24 (1.04-1.44)	0.89 (0.73-1.06)	1.70 (1.47-1.93)
0_11	0.65 (0.38-1.04)	0.31 (0.13-0.75)	3.99 (3.21-4.89)	1.62 (1.15-2.23)	0.76 (0.44-1.20)	1.36 (0.93-1.91)	1.44 (1.00-2.02)	0.58 (0.32-0.97)	1.03 (0.66-1.53)
12-23	1.14 (0.75-1.64)	1.45 (0.99-2.04)	12.55 (11.13-14.09)	1.50 (1.05-2.09)	2.03 (0.49-2.69)	2.77 (2.15-3.52)	1.88 (1.36-2.52)	1.04 (0.67-1.54)	1.72 (1.23-2.34)
24-59	0.57 (0.40-0.78)	0.97 (0.76-1.23)	7.17 (6.57-7.82)	1.21 (0.98-1.49)	0.84 (0.64-1.07)	2.12 (1.18-2.47)	0.93 (0.77-1.24)	0.94 (0.74-1.20)	1.91 (1.61-2.25)
Region 8									
AdjR (95% CI)	6.75 (6.28-7.23)	1.35(1.14-1.57)	1.52 (1.28-1.75)	1.91 (1.64-2.17)	0.93 (0.75-1.11)	2.01 (1.74-2.27)	2.77 (2.45-3.09)	3.01(2.69-3.32)	4.22 (3.83-4.62)
0-11	7.91 (6.81-9.11)	1.50 (1.03-2.11)	1.25 (0.83-1.80)	1.61 (1.14-2.22)	1.03 (0.66-1.54)	2.12 (1.57-2.79)	2.56 (1.94-3.32)	3.85 (3.09-4.75)	4.76 (3.89-5.76)
12-23	13.87 (12.38-15.49)	1.73 (1.23-2.39)	1.61 (1.13-2.23)	3.16 (2.47-3.97)	1.47 (1.02-2.06)	3.02 (2.36-3.81)	3.34 (2.62-4.19)	3.92 (3.14-4.83)	6.60 (5.57-7.77)
24-59	3.76 (3.30-4.26)	1.16 (0.92-1.44)	1.58 (1 .27-1.93)	1.56 (1.26-1.91)	0.70 (0.51-0.94)	1.60 (1.30-1.95)	2.64 (2.24-3.10)	2.37 (2.03-2.75)	3.17 (2.74-3.65)

AdjR = Age-adjusted prevalence rate of consultations for asthma, expressed per 1000 childhood population ; 95%CI= confidence interval at 95%.

* *D*irectly standardized rates method is unreliable with small number (number of total observed events should be >= 25)

Additional File 1 : Continued

Age group	2004	2005	2006	2007	2008	2009	2010	2011	2012
Region 9									
AdjR (95% CI)	4.35 (4.09-4.60)	2.44 (2.25-2.62)	5.74 (5.45-6.03)	5.58 (5.30-5.86)	10.50 (10.13-10.87)	7.75 (7.43-8.07)	8.70 (8.35-9.06)	7.43 (7.10-7.76)	8.38 (8.03-8.74)
0-11	4.50 (3.96-5.09)	1.68 (1.36-2.05)	3.94 (3.44-4.48)	5.67 (5.08-6.32)	11.37 (10.53-12.27)	5.78 (5.19-6.43)	5.83 (4.22-6.49)	5.60 (5.00-6.25)	7.00 (6.32-7.74)
12-23	5.86 (5.24-6.54)	2.97 (2.54-3.47)	8.63 (7.89-9.43)	6.12 (5.50-6.79)	17.80 (16.73-18.91)	11.92 (11.06-12.83)	11.12 (10.26-12.03)	10.29 (9.47-11.16)	11.52 (10.64-12.46)
24-59	3.52 (3.24-3.81)	2.55 (2.32-2.80)	5.20 (4.86-5.56)	5.26 (4.93-5.62)	7.79 (7.39-8.22)	7.03 (6.64-7.42)	8.94 (8.48-9.42)	6.92 (6.52-7.35)	7.51 (7.10-7.94)
Region 10									
AdjR (95% CI)	6.82 (6.43-7.20)	7.36 (6.97-7.74)	12.85 (12.32-13.38)	12.93 (12.40-13.45)	12.04 (11.54-12.54)	13.91 (13.37-14.44)	16.34 (15.76-16.93)	17.19 (16.60-17.79)	20.91 (20.26-21.57)
0-11	4.50 (3.84-5.25)	6.48 (5.65-7.39)	8.96 (7.99-10.01)	9.72 (8.73-10.80)	10.81 (9.77-11.94)	11.86 (10.78-13.03)	15.02 (13.81-16.30)	17.76 (16.44-19.15)	18.33 (17.00-19.73)
12-23	8.92 (7.96-9.97)	9.51 (8.50-10.61)	20.01 (18.55-21.55)	17.34 (16 .00-18.76)	17.25 (15.92-18.66)	18.63 (17.27-20.08)	19.09 (17.71-20.53)	22.65 (21.16-24.22)	28.71 (27.04-30.47)
24-59	6.88 (6.40-7.39)	6.95 (6.50-7.42)	11.81 (11.17-12.48)	12.55 (11.89-13.23)	10.77 (10.17-11.39)	13.05 (12.39-13.73)	15.89 (15.15-16.66)	15.26 (14.54-16.01)	19.24 (18.43-20.08)
Region 11									
AdjR (95% CI)	1.74 (1.44-2.04)	3.48 (3.06-3.89)	2.88 (2.47-3.29)	0.92 (0.69-1.15)	0.98 (0.74-1.21)	1.13 (0.88-1.38)	1.62 (1.31-1.92)	1.13 (0.87-1.38)	1.10 (0.85-1.35)
0-11	1.64 (1.08-2.39)	1.60 (1.01-2.40)	0.41 (0.15-0.90)	0.54 (0.23-1.06)	0.20 (0.04-0.59)	0.47 (0.19-0.96)	1.03 (0.58-1.70)	2.03 (1.37-2.89)	0.48 (0.19-0.99)
12-23	1.80 (1.19-2.60)	1.54 (0.96-2.33)	2.96 (2.14-3.99)	1.09 (0.62-1.76)	0.54 (0.23-1.06)	1.20 (0.71-1.90)	3.24 (2.38-4.31)	1.36 (0.83-2.10)	1.45 (0.90-2.22)
24-59	1.76 (1.38-2.20)	4.93 (4.31-5.62)	3.79 (3.19-4.47)	1.00 (0.71-1.37)	1.44 (1.09-1.87)	1.36 (1.02-1.78)	1.22 (0.89-1.64)	0.69 (0.45-1.02)	1.20 (0.88-1.60)
Region 12									
AdjR (95% CI)	0.41 (0.24-0.58)	0.67 (0.46-0.89)	1.89 (1.51-2.27)	1.82 (1.45-2.19)	2.00 (1.62-2.38)	1.29 (0.98-1.59)	1.54 (1.19-1.89)	1.78 (1.40-2.15)	4.72 (4.12-5.32)
0-11	0.41 (0.13-0.96)	0.48 (0.15-1.11)	1.50 (0.86-2.44)	1.38 (0.77-2.28)	1.19 (0.63-2.03)	0.90 (0.43-0.66)	0.85 (0.39-1.62)	0.65 (0.26-1.35)	3.96 (2.84-5.37)
12-23	0.44 (0.14-1.02)	0.48 (0.16-1.12)	2.17 (1.37-3.25)	1.95 (1.20-2.97)	1.75 (1.05-2.73)	1.45 (0.83-2.40)	1.24 (0.66-2.12)	1.97 (1.22-3.02)	5.24 (3.93-6.83)
24-59	0.39 (0.20-0.69)	0.81 (0.53-1.18)	1.93 (1.46-2.50)	1.93 (1.47-2.50)	2.38 (1.86-2.99)	1.36 (0.98-1.84)	1.89 (1.42-2.47)	2.10 (1.60-2.71)	4.81(4.05-5.68)
Region 13									
AdjR (95% CI)	0.44 (0.32-0.55)	2.69 (2.38-3.00)	0.96 (0.77-1.14)	1.81 (1.56-2.06)	0.61 (0.47-0.75)	0.36 (0.25-0.47)	0.50 (0.37-0.63)	0.88 (0.70-1.05)	1.32 (1.11-1.53)
0-11	0.74 (0.43-1.19)	1.82 (1.29-2.50)	1.08 (0.68-1.62)	1.97 (1.43-2.65)	0.41 (0.19-0.77)	0.89 (0.54-1.37)	0.37 (0.16-0.73)	0.54 (0.28-0.95)	0.93 (4.05-5.68)
12-23	0.56 (0.29-0.98)	3.95 (3.14-4.90)	1.37 (0.91-1.96)	2.12 (1.55-2.83)	1.00 (0.63-1.52)	0.22 (0.07-0.52)	1.22 (0.80-1.79)	1.46 (1.00-2.06)	2.52 (0.57-1.44)
24-59	0.30 (0.20-0.44)	2.57 (2.21-2.97)	0.79 (0.59-1.03)	1.66 (1.37-1.99)	0.55 (0.39-0.75)	0.24 (0.14-0.38)	0.32 (0.20-0.49)	0.80 (0.60-1.05)	1.07 (1.90-3.30)
Region 14									
AdjR (95% CI)	1.21 (1.02-1.41)	2.13 (1.85-2.41)	2.77 (2.46-3.09)	2.36 (2.07-2.65)	4.03 (3.66-4.40)	4.54 (4.15-4.93)	5.29 (4.85-5.72)	6.56 (6.08-7.04)	8.72 (8.17-9.28)
0-11	0.91 (0.56-1.41)	3.74 (2.94-4.68)	4.81 (3.92-5.85)	4.19 (3.37-5.16)	6.38 (5.36-7.54)	7.97 (0.68-9.25)	9.12 (7.87-10.58)	13.62 (12.09-15.28)	14.62 (13.04-16.35)
12-23	2.17 (1.60-2.89)	3.20 (2.47-4.08)	4.20 (3.36-5.18)	3.31 (2.58-4.18)	6.58 (5.54-7.76)	7.07 (6.00-8.28)	9.18 (7.93-10.58)	9.41 (8.15-10.81)	14.89 (13.29-16.64)
24-59	1.01 (0.81-1.25)	1.29 (1.03-1.60)	1.69 (1.39-2.04)	1.49 (1.22-1.82)	2.50 (2.14-2.90)	2.68 (2.31-3.09	2.88 (2.47-3.33)	3.47 (3.03-3.96)	4.96 (4.45-5.52)
Region 15									
AdjR (95% CI)	5.47 (4.79-6.14)	2.76(2.28-3.24)	0.91 (0.62-1.19)	4.16 (3.56-4.77)	2.58 (2.11-3.05)	4.02 (3.44-4.61)	3.53 (2.94-4.13)	7.11 (6.26-7.95)	3.40 (2.81-3.99)
0-11	0.32 (0.07-0.94)	1.99 (1.16-3.19)	0.69 (0.25-1.51)	7.96 (6.20-10.08)	2.29 (1.40-3.54)	7.69 (7.97-9.75)	6.70 (5.01-8.79)	3.70 (2.48-5.32)	3.64 (2.40-5.29)
12-23	1.71 (0.96-2.82)	3.42 (2.29-4.91)	0.93 (0.40-1.83)	4.90 (3.53-6.62)	1.27 (0.64-2.28)	4.46 (3.17-6.09)	3.00 (1.90-4.49)	9.94 (7.84-12.42)	6.80 (5.05-8.96)
24-59	8.31 (7.28-9.45)	2.80 (2.23-3.48)	0.97 (0.63-1.43)	2.71 (2.12-3.41)	3.09 (2.46-3.82)	2.71 (2.12-3.40)	2.69 (2.06-3.45)	7.30 (6.23-8.50)	2.25 (1.68-2.95)
Region 16									
AdjR (95% CI)	1.48 (1.29-1.68)	1.63 (1.41-1.84)	1.91 (1.68-2.14)	2.81 (2.53-3.10)	2.45 (2.19-2.70)	2.53 (2.27-2.78)	2.15 (1.91-2.40)	4.33(3.99-4.66)	4.72 (4.38-5.06)
0-11	1.01 (0.69-1.43)	2.89 (2.28-3.60)	2.14 (1.63-2.76)	3.14 (2.52-3.86)	2.30 (1.78-2.92)	2.46 (1.93-3.10)	2.09 (1.60-2.69)	2.92 (2.35-3.59)	1.64 (1.23-2.16)
12-23	1.95 (1.48-2.52)	1.62 (1.17-2.17)	2.12 (1.61-2.73)	4.27 (3.55-5.10)	4.21 (3.50-5.03)	4.14 (3.43-4.94)	3.36 (2.72-4.11)	7.87 (6.92-8.92)	6.77 (5.89-7.74)
24-59	1.49 (1.28-1.76)	1.20 (0.98-1.45)	1.76 (1.48-2.07)	2.20 (1.87-2.57)	1.89 (1.61-2.21)	2.00 (1.71-2.32)	1.76 (1.80-2.08)	3.60 (3.20-4.02)	3.17 (4.63-5.56)
