# Supplementary material for: Secular trends in consultations for asthma in early childhood, the 16 administrative regions of Morocco, 2004–2012
Source: BMC Public Health. 2015 Sep 17;15:905. doi: 10.1186/s12889-015-2262-8 (PMC4574130; doi:10.1186/s12889-015-2262-8)
Supplement: Additional file 2: — Direct method for age-adjusted prevalence rate of consultations for asthma in under-5 children, rural areas, Morocco: 9-year trends (2004-2012). (PDF 44 kb) [file 12889_2015_2262_MOESM2_ESM.pdf]

Additional File 2 : Direct method for age-adjusted prevalence rate of consultations for asthma in under-5 children, rural areas, Morocco : 9-year trends (2004-2012)

Age group (months)	2004	2005	2006	2007	2008	2009	2010	2011	2012
Region 1									
AdjR (95% CI)	0	0	0	0	0	0	0	0	0
0-11	0 (0-4.46)	0 (0-3.14)	0 (0.-2.68)	0 (0-2.29)	0 (0-1.98)	0 (0-1.70)	0 (0-1.73)	0 (0-1.43)	0 (0-1.85)
12-23	0 (0-4.93))	0 (0-3.19)	0 (0-2.72)	0 (0-0.32)	0 (0-2.01)	0 (0-1.72)	0(0-1.76)	0 (0-1.45)	0 (0-1.88)
24-59	0 (0-2.00)	0 (0-0.95)	0 (0-1.88)	0 (0-1.60)	0 (0-1.33)	0 (0-1.17)	0 (0-1.30)	0 (0-1.11)	0 (0-1.60)
Region 2									
AdjR (95% CI)	0	*	*	0	0	0	*	0	*
0-11	0 (0-19.21)	0 (0-7.75)	0 (0-7.53)	0 (0-7.36)	0 (0-7.32)	0 (0-7.20)	0 (0-17.48)	0 (0-17.48)	0 (0-20.72)
12-23	0 (0-21.2)	0 (0-7.87)	0 (0-7.56)	0 (0-7.48)	0 (0-7.44)	0 (0-7.32)	0 (0-17.82)	0 (0-17.73)	11.43 (1.38-41.28)
24-59	0 (0-9.94)	2.07 (0.43-6.05)	2.53 (0.31-9.16)	0(0-4.55)	0 (0-4.36)	0 (0-4.39)	2.80 (0.07-15.61)	0 (0-10.79)	18.94 (6.44.20)
Region 3									
AdjR (95% CI)	0.85 (0.46-1.25)	*	3.08 (2.26-3.89)	2.03 (1.38-2.69)	*	*	*	*	2.66 (1.84-3.48)
0-11	0.22 (0.01- 1.24)	0 (0-0.85)	0.68 (0.14-2.00)	4.32 (2.60-6.74)	1.61 (0.65- 3.32)	0.46 (0.06-1.67)	0.71 (0.15-2.08)	0 (0-1.87)	1.68 (0.68-3.47)
12-23	0.18 (0.38-2.75)	0.70 (0.14-2.04)	1.16 (0.38-2.70)	1.38 (0.51-3.01)	0.47 (0.06-1.69)	0.23 (0.01-0.30)	0.48 (0.06-1.74)	0.70 (0.15-2.10)	2.69 (1.34-4.81)
24_59	0.99 (0.51-1.74)	1.12 (0.62-1.89)	5.01 (3.67-6.68)	1.29 (0.67-2.25)	0.94 (0.43-1.78)	0.84 (0.36-1.66)	0.95 (0.41-1.88)	0.37 (0.08-1.07)	3.09 (1.96-4.64)
Region 4									
AdjR (95% CI)	0.71 (0.60-0.82)	0.78 (0.66-0.89)	1.60 (1.43-1.78)	0.86 (0.73-0.99)	1.03 (0.89-1.17)	0.99 (0.85-1.13)	1.97 (1.76-2.17)	1.20 (1.05-1.36)	4.27 (3.95-4.60)
0-11	0,87 (0.62-1.18)	0.58 (0.38-0.85)	0.65 (0.44-0.94)	0.72 (0.49-1.01)	1.34 (0.84-1.50)	1.07 (0.78-1.42)	1.97 (1.57-2.44)	1.41 (1.08-1.81)	5.36 (4.71-6.08)
12-23	0,98 (0.71-1.33)	1.38 (1.06-1.77)	1.88 (1.50-2.34)	1.53 (1.18-1.94)	1.24 (0.93-1.62)	1.64 (1.28-2.07)	2.23 (1.79-2.74)	1.62 (1.26-2.05)	7.79 (6.75-8.93)
24-59	0,54 (0.42-0.69)	0.63 (0.51-0.77)	1.86 (1.62-2.13)	0.66 (0.52-0.83)	0.90 (0.74-1.09)	0.72 (0.57-0.89)	1.87 (1.61-2.15)	0.71 (0.79-1.19)	2.55 (2.24-2.88)
Region 5									
AdjR (95% CI)	0.21 (0.13-0.29)	0.80 (0.65-0.96)	0.30 (0.21-0.40)	1.38 (1.18-1.59)	0.32 (0.22-0.41)	2.57(2.29-2.85)	4.74 (4.33-5.14)	1.20 (0.98-1.42)	0.93 (0.72-1.13)
0-11	0.19 (0.06-0.44)	0.58 (0.32-0.97)	0.29 (0.12-0.59)	1.10 (0.72-1.60)	0.08 (0.01-0.30)	4.60 (3.79-5.53)	1.79 (1.28-2.43)	0.46 (0.21-0.88)	0.53 (0.26-0.94)
12-23	0.48 (0.25-0.84)	0.59 (0.32-0.98)	0.37 (0.17-0.71)	2.19 (1.64-2.86)	0.63 (0.35-1.03)	4.63 (3.81-5.57)	2.48 (1.88-3.22)	1.78 (1.23-2.48)	2.40 (1.66-3.36)
24-59	0,14 (0.07-0.25)	0.94 (0.74-1.17)	0.29 (0.18-0.44)	1.23 (0.99-1.50)	0.29 (0.19-0.44)	1.32 (1.07-1.60)	6.35 (5.76-6.97)	1.25 (0.98-1.58)	0.60 (0.41-0.83)
Region 6									
AdjR (95% CI)	7.23 (6.70-7.76)	6.20 (5.72-6.68)	2.96 (2.63-3.30)	2.95 (2.62-3.29)	7.34 (6.81-7.87)	2.39 (2.09-2.69)	8.54 (7.95-9.14)	6.14 (5.63-6.63)	24.67 (23.61-25.73)
0-11	7.89 (6.70-9.23)	8.99 (7.73-10.43)	3.06 (2.33-3.95)	5.29 (4.32-6.43)	13.80 (12.18-15.58)	3.41 (2.62-4.35)	10.70 (9.24-12.32)	9.21 (7.86-10.72)	2.86 (2.14-3.75)
12-23	13.70 (12.07-15.49)	10.91 (9.48-12.51)	5.38 (4.38-6.53)	4.64 (3.72-5.71)	14.44 (12.77-16.28)	4.16 (3.28-5.20)	16.10 (14.29-18.08)	10.25 (8.82-11.86)	6.68 (5.41-8.15)
24-59	5.11 (4.54-5.73)	3.95 (3.48-4.47)	2.21 (1.86-2.62)	1.74 (1.43-2.11)	3.27 (2.84-3.75)	1.56 (1.26-1.90)	5.64 (5.03-6.31)	3.98 (3.48-4.53)	36.61 (34.97-38.31)
Region 7									
AdjR (95% CI)	0.89 (0.76-1.02)	1.18 (1.04-1.32)	0.86 (0.74-0.99)	1.44 (1.28-1.60)	1.60 (1.43-1.76)	0.98 (0.85-1.12)	1.34 (1.18-1.51)	1.39 (1.23-1.56)	4.79 (4.49-5.10)
0-11	0.80 (0.56-1.09)	1.69 (1.33-2.10)	1.05 (0.78-1.39)	2.07 (1.68-2.53)	1.73 (1.37-2.15)	1.45 (0.86-1.50)	1.56 (0.86-1.52)	1.37 (1.04-1.76)	6.69 (5.96-7.48)
12-23	0.97 (0.70-1.31)	1.60 (1.26-2.01)	1.11 (0.83-1.46)	1.84 (1.47-2.28)	2.22 (1.81-2.70)	1.71 (1.36-2.14)	1.99 (1.59-2.47)	2.68 (2.21-3.22)	7.54 (6.76-8.39)
24-59	0.89 (0.73-1.09)	0.84 (0.70-0.99)	0.70 (0.56-0.87)	1.06 (0.88-1.26)	1.32 (1.12-1.54)	0.66 (0.52-0.82)	1.17 (0.98-1.40)	0.93 (0.75-1.14)	3.08 (2.75-3.44)
Region 8									
AdjR (95% CI)	0.38 (6.23-0.52)	1.07(0.84-1.31)	0.87 (0.65-1.10)	3.98 (3.51-4 .45)	1.03 (0.79-1.27)	1.77 (1.44-2.09)	0.69 (0.48-0.90)	0.72 (0.50-0.94)	1.75 (1.42-2.07)
0-11	0.26 (0.07-0.66)	1.45 (0.90-2.22)	0.28 (0.08-0.72)	6.66 (5.38-8.15)	0.44 (0.16-0.95)	1.12 (0.62-1.84)	0.40 (0.13-0.93)	0.64 (0.28-1.27)	2.24 (1.52-3.19)
12-23	0.21 (0.04-0.61)	1.69 (1.08-2.51)	1.42 (0.87-2.20)	6.05 (4.82-7.49)	1.63 (1.02-2.47)	2.34 (1.59-3.32)	0.89 (0.44-1.59)	0.98 (0.51-1.71)	3.23 (2.35-4.34)
24-59	0.48 (0.28-0.74)	0.74 (0.51-1.03)	0.89 (0.62-1.24)	2.36 (1.91-2.89)	1.03 (0.74-1.39)	1.80 (1.40-2.27)	0.72 (0.46-1.06)	0.66 (0.41-0.99)	1.07 (0.76-1.48)

AdjR = Age-adjusted prevalence rate of consultations for asthma, expressed per 1000 childhood population ; 95%CI= confidence interval at 95%.
**** D*irectly standardized rates method is unreliable with small number (number of total observed events should be >= 25)**

Additional File 2 : Continued

Age group	2004	2005	2006	2007	2008	2009	2010	2011	2012
Region 9									
AdjR (95% CI)	2.72 (1.74-3.70)	*	1.92 (1.49-2.34)	0.87 (0.59-1.15)	1.03 (0.73-1.32)	1.16 (0.85-1.47)	*	*	*
0-11	4.69 (2.94-7.10)	0.89 (0.29-2.07)	1.02 (0.37-2.21)	0.97 (0.36-2.11)	0.47 (0.10-1.37)	0.15 (0.004-0.83)	0 (0-0.77)	0.61 (5.00-6.25)	0 (0-0.73)
12-23	3.12 (1.71-5.24)	0.72 (0.20-1.85)	1.89 (0.94-3.39)	1.31 (0.57-2.58)	1.47 (0.87-3.12)	0.91 (0.34-1.97)	0 (0-0.79)	0 (0-0.77)	0.40 (0.05-1.44)
24-59	2.25 (1.16-3.93)	0.59 (0.30-1.06)	2.10 (1.60-2.70)	0.76 (0.48-1.15)	1.00 (0.68-1.41)	1.42 (1.04-1.89)	0.27 (0.10-0.58)	0.05 (0.001-0.25)	0.55 (0.29-0.96)
Region 10									
AdjR (95% CI)	3.05 (2.53-3.57)	1.58 (1.24-1.92)	1.27 (0.95-1.60)	1.11 (0.81-1.42)	3.08 (2.58-3.59)	1.75 (1.36-2.13)	1.59 (1.19-1.97)	*	1.38 (1.01-1.76)
0-11	0.99 (0.45-1.87)	1.23 (0.63-2.14)	0.71 (0.29-1.46)	0.50 (0.16-1.18)	5.41 (4.05-7.08)	1.94 (1.17-3.03)	1.69 (0.95-2.79)	0.11 (0.003-0.64)	0.57 (0.18-1.33)
12-23	1.72 (0.94-2.89)	1.77 (1.03-2.83)	2.16 (1.34-3.31)	2.05 (1.25-3.16)	4.15 (2.96-5.64)	3.94 (2.79-5.40)	1.72 (0.96-2.83)	0.35 (0.07.1.02)	2.42 (1.50-3.70)
24-59	4.29 (3.52-5.18)	1.64 (1.23-2.15)	1.16 (0.78-1.65)	1.00 (0.65-1.46)	1.84 (1.36-2.43)	0.88 (0.56-1.32)	1.49 (1.03-2.08)	0.64 (0.35-1.07)	1.31 (0.86-1.90)
Region 11									
AdjR (95% CI)	2.92 (2.63-3.21)	1.23 (1.05-1.41)	0.89 (0.74-1.04)	2.04 (1.81-2.27)	0.48 (0.36-0.58)	1.53 (1.33-1.73)	2.22 (1.97-2.47)	2.30 (2.05-2.57)	2.00 (1.75-2.24)
0-11	2.04 (1.55-2.63)	0.54 (0.30-0.89)	0.93 (0.61-1.36)	2.80 (2.21-3.49)	0.04 (0.001-0.20)	1.29 (0.901-1.78)	2.90 (2.28-3.63)	3.43 (2.75-4.21)	1.99 (1.49-2.59)
12-23	4.24 (3.46-5.15)	1.31 (0.92-1.82)	1.67 (1.22-2.22)	3.13 (2.50-3.86)	0.90 (058-1.32)	2.76 (2.17-3.45)	3.60 (2.91-4.41)	3.48 (2.80-4.28)	2.24 (1.71-2.88)
24-59	2.79 (2.44-3.18)	1.40 (1.17-1.67)	0.64 (0.49-0.83)	1.50 (1.26-1.77)	0.47 (0.34-0.63)	1.36 (1.01-1.48)	1.61 (1.34-1.91)	1.63 (1.36-1.94)	1.93 (1.63-2.26)
Region 12									
AdjR (95% CI)	1.08 (0.87-1.28)	0.58 (0.44-0.72)	0.89 (0.70-1.07)	1.97 (1.69-2.24)	1.54 (1.30-1.78)	1.17 (0.96-1.38)	3.25 (2.88-3.62)	0.67 (0.50-0.84)	1.66 (1.40-1.93)
0-11	0.20 (0.05-0.51)	0.05 (0.001-0.27)	0.38 (0.16-0.75)	0.33 (0.13-0.68)	1.34 (0.89-1.94)	0.58 (0.30-1.00)	2.69 (2.02-3.52)	0.56 (0.28-1.01)	0.87 (0.52-1.38)
12-23	0.76 (0.42-1.28)	1.93 (1.38-2.63)	0.91 (0.55-1.43)	4.12 (3.30-5.09)	3.31 (2.57-4.19)	1.80 (1.27-2.49)	4.90 (3.96-5.99)	0.73 (0.40-1.22)	2.85 (2.16-3.68)
24-59	1.49 (1.20-1.84)	0.30 (0.19-0.46)	1.06 (0.81-1.35)	1.80 (1.47-2.18)	1.00 (0.76-1.29)	1.16 (0.90-1.47)	2.87 (2.43-3.37)	0.69 (0.48-0.96)	1.53 (1.21-1.92)
Region 13									
AdjR (95% CI)	1.00 (0.80-1.20)	1.57 (1.32-1.81)	2.13 (1.84-2.43)	0.79 (0.61-0.97)	0.67 (0.50-0.83)	1.14 (0.92-1.35)	2.31 (1.98-2.63)	1.20 (0.95-1.45)	1.39 (1.13-1.67)
0-11	2.08 (1.53-2.76)	3.25 (2.54-4.09)	2.93 (2.26-3.73)	1.17 (0.77-1.72)	0.92 (0.56-1.42)	0.79 (0.46-1.26)	2.47 (1.83-3.25)	0.79 (0.44-1.31)	1.00 (0.61-1.54)
12-23	1.05 (0.66-1.57)	2.61 (1.98-3.38)	1.05 (0.67-1.58)	0.41 (0.19-0.78)	0.51 (0.26-0.92)	1.37 (0.92-1.96)	2.91 (2.21-3.76)	1.34 (0.87-1.98)	1.83 (1.28-2.53)
24-59	0.52 (0.35-0.75)	0.43 (0.29-0.61)	2.25 (1.85-2.70)	0.79 (0.56-1.08)	0.62 (0.43-0.88)	1.18 (0.90-1.53)	1.99 (1.59-2.45)	1.31 (0.97-1.73)	1.38 (1.02-1.81)
Region 14									
AdjR (95% CI)	0.15 (0.03-0.27)	*	0.29 (0.14-0.45)	0.83 (0.57-1.09)	0.27 (0.12-0.42)	1.82 (1.44-2.20)	0.53 (0.31-0.75)	2.42 (1.95-2.88)	3.81 (3.22-4.41)
0-11	0.11 (0.003-0.62)	0.84 (0.36-1.66)	0.42 (0.11-1.07)	0 (0-0.38)	0.31 (0.06-0.92)	0.86 (2.72-5.32)	0.55 (1.18-1.29)	2.21 (1.35-3.41)	0.51(0.24-0.93)
12-23	0 (0 -0.45)	0 (0-0.39)	0.53 (0.17-1.23)	0.21 (0.03-0.76)	0.42 (0.12-1.09)	1.17 (0.58-2.09)	0.34 (0.07-0.99)	1.57 (0.86-2.63)	1.51 (0.83-2.53)
24-59	0.21 (0.07-0.49)	0.15 (0.04-0.37)	0.18 (0.06-0.41)	1.30 (0.91-1.79)	0.21 (0.08-0.45)	1.36 (0.978-1.86)	0.59 (0.33-0.97)	2.76 (2.14-3.49)	5.65 (4.74-6.67)
Region 15									
AdjR (95% CI)	2.15 (1.93-2.38)	1.61 (1.41-1.81)	1.01 (0.85-1.17)	1.05 (0.88-1.21)	1.16 (0.98-1.33)	2.63 (2.36-2.89)	1.13 (0.95-1.30)	3.08 (2.79-3.37)	1.52 (1.32-1.72)
0-11	0.86 (0.58-1.24)	1.23 (0.86-1.69)	1.92 (1.46-2.48)	1.44 (1.03-1.95)	2.18 (1.67-2.80)	4.57 (3.81-5.43)	1.41 (1.00-1.92)	2.56 (2.00-3.23)	2.19 (1.68-2.80)
12-23	2.42 (1.90 -3.04)	3.10 (2.50-3.80)	0.94 (0.63-1.36)	0.93 (0.60-1.36)	0.62 (0.36-0.99)	3.76 (3.07-4.56)	1.68 (1.23-2.25)	5.79 (4.92-6.77)	3.21 (2.58-3.94)
24-59	2.46 (2.16-2.80)	1.28 (1.07-1.53)	0.76 (0.59-0.96)	0.97 (0.78-1.19)	1.01 (0.82-1.24)	1.71 (1.45-2.00)	0.88 (0.69-1.10)	2.44 (2.11-2.80)	0.82 (0.63-1.04)
Region 16									
AdjR (95% CI)	0.11 (0.09-0.13)	1.58 (1.37-1.79)	1.81 (1.58-2.04)	3.34 (3.03-3.65)	2.98 (2.68-3.27)	2.59 (2.32-2.87)	4.30 (3.931-4.67)	2.75(2.47-3.03)	3.55 (3.25-3.86)
0-11	0.21 (0.16-0.26)	2.68 (2..09-3.38)	2.60 (2.03-3.29)	4.95 (4.15-5.26)	4.35 (3.60-5.21)	4.10 (3.38-4.93)	5.09 (4.26-6.04)	4.92 (4.14-5.80)	4.48 (3.73-5.33)
12-23	0.14 (0.10-0.18)	2.38 (1.82-3.05)	2.98 (2.36-3.72)	4.40 (3.64-5.26)	4.42 (3.66-5.28)	4.13 (3.39-4.97)	6.79 (5.81-7.88)	3.94 (3.24-4.74)	6.03 (5.15-7.01)
24-59	0.07 (0.05-0.09)	0.93 (0.75-1.15)	1.13 (0.90-1.40)	2.42 (2.08-2.79)	2.00 (1.70-2.34)	1.54 (1.28-1.84)	3.17 (2.76-3.62)	1.58 (1.31-1.89)	2.37 (2.08-2.70)

AdjR = Age-adjusted prevalence rate of consultations for asthma, expressed per 1000 childhood population ; 95%CI= confidence interval at 95%.
*** *D*irectly standardized rates method is unreliable with small number (number of total observed events should be >= 25)**
